# Dual roles of chromatin remodeling protein BRG1 in angiotensin II-induced endothelial–mesenchymal transition

**DOI:** 10.1038/s41419-020-02744-y

**Published:** 2020-07-18

**Authors:** Zilong Li, Xiaochen Kong, Yuanyuan Zhang, Yangxi Zhang, Liming Yu, Junli Guo, Yong Xu

**Affiliations:** 1 Key Laboratory of Targeted Intervention of Cardiovascular Disease and Collaborative Innovation Center for Cardiovascular Translational Medicine, Department of Pathophysiology, Nanjing Medical University, Nanjing, China; 2 Institute of Biomedical Research, Liaocheng University, Liaocheng, China; 3 Department of Endocrinology, Affiliated Nanjing Municipal Hospital of Nanjing Medical University, Nanjing, China; 4 Hainan Provincial Key Laboratory for Tropical Cardiovascular Diseases Research and Key Laboratory of Emergency and Trauma of Ministry of Education, Institute of Cardiovascular Research of the First Affiliated Hospital, Hainan Medical University, Haikou, China

**Keywords:** Epigenetics, Transcription, Cardiovascular diseases

## Abstract

Endothelial–mesenchymal transition (EndMT) is considered one of the processes underlying tissue fibrosis by contributing to the pool of myofibroblasts. In the present study, we investigated the epigenetic mechanism whereby angiotensin II (Ang II) regulates EndMT to promote cardiac fibrosis focusing on the role of chromatin remodeling protein BRG1. BRG1 knockdown or inhibition attenuated Ang II-induced EndMT, as evidenced by down-regulation of *CDH5*, an endothelial marker, and up-regulation of *COL1A2*, a mesenchymal marker, in cultured vascular endothelial cells. On the one hand, BRG1 interacted with and was recruited by Sp1 to the *SNAI2* (encoding SLUG) promoter to activate *SNAI2* transcription in response to Ang II stimulation. Once activated, SLUG bound to the *CDH5* promoter to repress *CDH5* transcription. On the other hand, BRG1 interacted with and was recruited by SRF to the *COL1A2* promoter to activate *COL1A2* transcription. Mechanistically, BRG1 evicted histones from the target promoters to facilitate the bindings of Sp1 and SRF. Finally, endothelial conditional BRG1 knockout mice (CKO) exhibited a reduction in cardiac fibrosis, compared to the wild type (WT) littermates, in response to chronic Ang II infusion. In conclusion, our data demonstrate that BRG1 is a key transcriptional coordinator programming Ang II-induced EndMT to contribute to cardiac fibrosis.

## Introduction

Cardiac fibrosis, like any other organ-specific fibrogenic response, can be considered a host defense mechanism that safeguards the physiological integrity of the heart to prevent myocardial rupture and circulatory failure in the event of cardiac injury^1^. Uncontrolled, excessive fibrogenesis or the failure to terminate fibrogenesis properly, however, leads to impairment of cardiac architecture, dampens heart function, and is a hallmark event in chronic heart failure^2^. It is generally agreed that myofibroblast cells, a unique cell type with dual abilities of contraction (hence the prefix “myo”) and laying down extracellular matrix proteins (a fibroblast-like behavior), are the primary mediator of cardiac fibrosis^3^. Gene signature wise, myofibroblasts are typically characterized by high levels of smooth muscle actin alpha (α-SMA), collagen type I and III, and periostin^4^. Because myofibroblasts are absent from the healthy myocardium, origins from which myofibroblasts arise during cardiac injury and fibrosis have been actively pursued and hotly debated with considerable amount of controversy. It can be argued that multiple lineages of cell types, including resident fibroblast cells, pericytes, myeloid cells, fibrocytes, and endothelial cells, contribute to the pool of mature myofibroblasts during cardiac fibrosis^5^. For instance, Zeisberg et al. exploiting a *Tie2*-Cre driven lineage tracing system, demonstrated that a large fraction of α-SMA-positive myofibroblasts detected in the scarring region of the murine infarct following acute myocardial ischemia (AMI) might originate from endothelial cells, likely through a process known as endothelial–mesenchymal transition (EndMT)^6^. Similarly, Wang et al. have reported that EndMT contributes to activation of myofibroblasts and cardiac fibrosis in mice exposed to chronic angiotensin II (Ang II) infusion, which is mediated by transglutaminase 2^7^. It has also been suggested that the sequence-specific transcription factor Ets-1 might be responsible, at least in part, for Ang II-induced EndMT and cardiac fibrosis in mice^8^. In cultured endothelial cells, treatment with Ang II stimulates the down-regulation of endothelial marker genes (e.g., *CDH5* encoding VE-Cadherin, *PECAM1* encoding CD31, and *VWF* encoding von Willebrand factor) and up-regulation of mesenchymal marker genes (e.g., *COL1A1*/*COL1A2* encoding collagen type I and *VIM* encoding vimentin) although the epigenetic mechanism is not completely understood^9,10^. EndMT and the related process epithelial–mesenchymal transition (EMT) are programmed by a host of transcription factors, among which the E-box-binding family of proteins including SNAIL, SLUG, and ZEB have been well studied^11^.

In mammalian cells, gene transcription is profoundly influenced by the epigenetic machinery, which includes histone/DNA modifying enzymes, non-coding regulatory RNAs, and chromatin remodeling proteins. Brahma-related gene 1 (BRG1) is the catalytic core of the mammalian SWI/SNF chromatin remodeling complex. BRG1 regulates gene transcription by utilizing its ATPase activity to mobilize nucleosomes and alter chromatin structure. Germline deletion of BRG1 results in developmental arrest in mice suggesting a role for BRG1 in embryogenesis^12^. Recent investigations have revealed key roles for BRG1 in the regulation of cardiovascular diseases. Hang et al. have reported that postnatal deletion of BRG1 in the myocardium attenuates the development of pathological cardiac hypertrophy in response to pressure overload in mice by skewing the expression of myosin heavy chain isoforms^13^. We have recently found that endothelial-specific BRG1 deficiency attenuates atherosclerosis^14^, abdominal aortic aneurysm^15^, and cardiac ischemia-reperfusion injury^16,17^ in mice. Here we report that BRG1 mediates Ang II-induced EndMT in cultured cells by directly activating *COL1A2* transcription and indirectly repressing *CDH5* transcription. More importantly, endothelial conditional knockout of BRG1 in mice attenuates EndMT and cardiac fibrosis in mice subjected to chronic Ang II infusion.

## Methods

### Cell culture, plasmids, and transient transfection

Immortalized human endothelial cells (EAhy926, ATCC) and HEK293 cells were maintained in DMEM supplemented with 10% fetal bovine serum (FBS, Hyclone). Human primary microvascular endothelial cells (HMVEC) were purchased from Lonza and maintained in EGM-2 media with supplements supplied by the vendor; three different batches of primary cells were used in this study as previously described^18^. Primary murine cardiac microvascular endothelial cells were isolated as previously described^19^. Angiotensin II was purchased from Sigma. SNAI2/SLUG promoter-luciferase constructs^20^, COL1A2 promoter-luciferase constructs^21^, BRG1 expression constructs^22^, SLUG expression constructs^23^, Sp1 expression constructs^24^, and SRF expression constructs^25^ have been previously described. PFI-3 was purchased from Selleck. Transient transfections were performed with Lipofectamine 2000. Luciferase activities were assayed 24–48 h after transfection using a luciferase reporter assay system (Promega) as previously described^26^.

### Animals

All animal experiments were reviewed and approved by the Ethics Committee on Humane Treatment of Laboratory Animals of Nanjing Medical University and were performed in accordance with the ethical standards laid down in the 1964 Declaration of Helsinki and its later amendments. The *Smarca4*-flox mice^27^ and the Cdh5-Cre mice^28^ were crossed to make the endothelial conditional BRG1 knockout mice (CKO). The mice were housed with a 12:12 h light–dark cycle at constant room temperature, fed standard rodent diet, and allowed at least one week of acclimation before the start of the experiments. Male, 8-week-old mice were induced to develop cardiac fibrosis by Angiotensin II (1 μg/kg/min) infusion for 4 consecutive weeks using subcutaneously implanted minipumps (Alzet 2004). One day prior to the sacrifice, the mice were anesthetized using isoflurane and heart functions were evaluated by echocardiography (GE Vivid 7 equipped with a 14-MHz phase array linear transducer, S12, allowing a 150 maximal sweep rate). The mice were sacrificed.

### Protein extraction and Western blot

Whole cell lysates were obtained by re-suspending cell pellets in RIPA buffer (50 mM Tris pH 7.4, 150 mM NaCl, 1% Triton X-100) with freshly added protease inhibitor (Roche) as previously described^18,29,30^. Nuclear proteins were extracted using the NE-PER Kit (Pierce) following manufacturer’s recommendation. Prior to immunoprecipitation, the lysates were treated with DNase I (NEB, M0303) at 37 °C for 30 min. Specific antibodies or pre-immune IgGs (PII) were added to and incubated with cell lysates overnight before being absorbed by Protein A/G-plus Agarose beads (Santa Cruz). Precipitated immune complex was released by boiling with 1X SDS electrophoresis sample buffer. Alternatively, FLAG-conjugated beads (M2, Sigma) were added to and incubated with lysates overnight. Precipitated immune complex was eluted with 3X FLAG peptide (Sigma). Western blot analyses were performed with anti-BRG1 (Santa Cruz, sc-10768), anti-collagen type I (Rockland, 600-401-103), anti-α-SMA (Sigma, A2547), anti-SLUG (Cell Signaling Technology, 9585), anti-VE-Cadherin (Cell Signaling Technology, 2158), anti-β-actin (Sigma, A2228), anti-MYC (Santa Cruz, sc-40), anti-FLAG (Sigma, F3165), anti-GFP (Proteintech, 50430-2), anti-Sp1 (Abcam, ab13370), and anti-SRF (Cell Signaling Technology, 5147) antibodies. For densitometrical quantification, densities of target proteins were normalized to those of β-actin. Data are expressed as relative protein levels compared to the control group which is arbitrarily set as 1.

### RNA isolation and real-time PCR

RNA was extracted with the RNeasy RNA isolation kit (Qiagen). Reverse transcriptase reactions were performed using a SuperScript First-strand Synthesis System (Invitrogen) as previously described^29,31,32^. Real-time PCR reactions were performed on an ABI Prism 7500 system with the following primers: human *CDH5*, 5′-TCACCTTCTGCGAGGATATGG-3′ and 5′-GAGTTGAGCACCGACACATC-3′; human *PECAM1*, 5′-CTGCTGACCCTTCTGCTCTGTTC-3′ and 5′-GGCAGGCTCTTCATGTCAACACT-3′; human *VWF*, 5′-TTGACGGGGAGGTGAATGTG-3′ and 5′-ATGTCTGCTTCAGGACCACG-3′; human *COL1A1*, 5′-TCTGCGACAACGGCAAGGTG-3′ and 5′-GACGCCGGTGGTTTCTTGGT-3′; human *COL1A2*, 5′-GTGGCAGTGATGGAAGTGTG-3′ and 5′-AGGACCAGCGTTACCAACAG-3′; human *VIM*, 5′-GAGAACTTTGCCGTTGAAGC-3′ and 5′-TCCAGCAGCTTCCTGTAGGT-3′; human *BRG1*, 5′-TCATGTTGGCGAGCTATTTCC-3′ and 5′-GGTTCCGAAGTCTCAACGATG-3′; human *SNAI2*, 5′-CACCATGCCGCGCTCCTTCCTGGTC-3′ and 5′-TCAGTGTACACAGCAGCCAGA-3′; human *SNAIL*, 5′-GAAAGGCCTTCAACTGCAAA-3′ and 5′-TGACATCTGAGTGGGTCTGG-3′; human *ZEB1*, 5′-GCACCTGAAGAGGACCAGAG-3′ and 5′-TGCATCTGGTGTTCCATTTT-3′; mouse *Cdh5*, 5′-TCAACGCATCTGTGCCAGAGAT-3′ and 5′-CACGATTTGGTACAAGACAGTG-3′; mouse *Pecam1*, 5′-GACTCACGCTGGTGCTCTATGC-3′ and 5′-TCAGTTGCTGCCCATTCTCA-3′; mouse *Col1a2*, 5′-GCCACCATTGATAGTCTCTCC-3′ and 5′-CACCCCAGCGAAGAACTCATA-3′; mouse *Vim*, 5′-CGGAAAGTGGAATCCTTGCA-3′ and 5′-CACATCGATCTGGACATGCTGT-3′; mouse *Snai2*, 5′-CGAACTGGACACACACACAG-3′ and 5′-AAAGGAGAGTGGAGTGGAGC-3′. Ct values of target genes were normalized to the Ct values of housekeekping control gene (18s, 5′-CGCGGTTCTATTTTGTTGGT-3′ and 5′-TCGTCTTCGAAACTCCGACT-3′ for both human and mouse genes) using the ΔΔCt method and expressed as relative mRNA expression levels compared to the control group which is arbitrarily set as 1.

### Chromatin immunoprecipitation (ChIP)

ChIP assays were performed essentially as described before^22,26,33–44^. In brief, chromatin in control and treated cells were cross-linked with 1% formaldehyde. Cells were incubated in lysis buffer (150 mM NaCl, 25 mM Tris pH 7.5, 1% Triton X-100, 0.1% SDS, 0.5% deoxycholate) supplemented with protease inhibitor tablet and PMSF. DNA was fragmented into ~200 bp pieces using a Branson 250 sonicator. Aliquots of lysates containing 200 μg of protein were used for each immunoprecipitation reaction with anti-BRG1 (Santa Cruz, sc-10768), anti-Sp1 (Abcam, ab13370), anti-SRF (Cell Signaling Technology, 5147), (Santa Cruz, sc-585), anti-SLUG (Cell Signaling Technology, 9585), anti-ZEB1 (Cell Signaling Technology, 3396), anti-SNAIL (Cell Signaling Technology, 3879), anti-acetyl H3 (Millipore, 06-599), anti-acetyl H4 (Millipore, 06-598), anti-histone H3 (Millipore, 06-755), or pre-immune IgG. For re-ChIP, immune complexes were eluted with the elution buffer (1% SDS, 100 mM NaCO_3_), diluted with the re-ChIP buffer (1% Triton X-100, 2 mM EDTA, 150 mM NaCl, 20 mM Tris pH 8.1), and subject to immunoprecipitation with a second antibody of interest.

### Histology

Histologic analyses were performed essentially as described before. Briefly, paraffin-embedded sections were stained with picrosirius red (Sigma-Aldrich) according to standard procedures. Pictures were taken using an Olympus IX-70 microscope (Olympus, Tokyo, Japan). Quantifications were performed with Image J by two independent assessors. For each animal, at least three slides with ~5 fields for each slide were included for quantification.

### Statistical analysis

One-way ANOVA with post-hoc Scheff´e analyses were performed by SPSS software (IBM SPSS v18.0, Chicago, IL, USA). Unless otherwise specified, values of *p* < 0.05 were considered statistically significant.

## Results

### BRG1 deficiency attenuates Ang II-induced EndMT in cultured cells

**Fig. 1:**
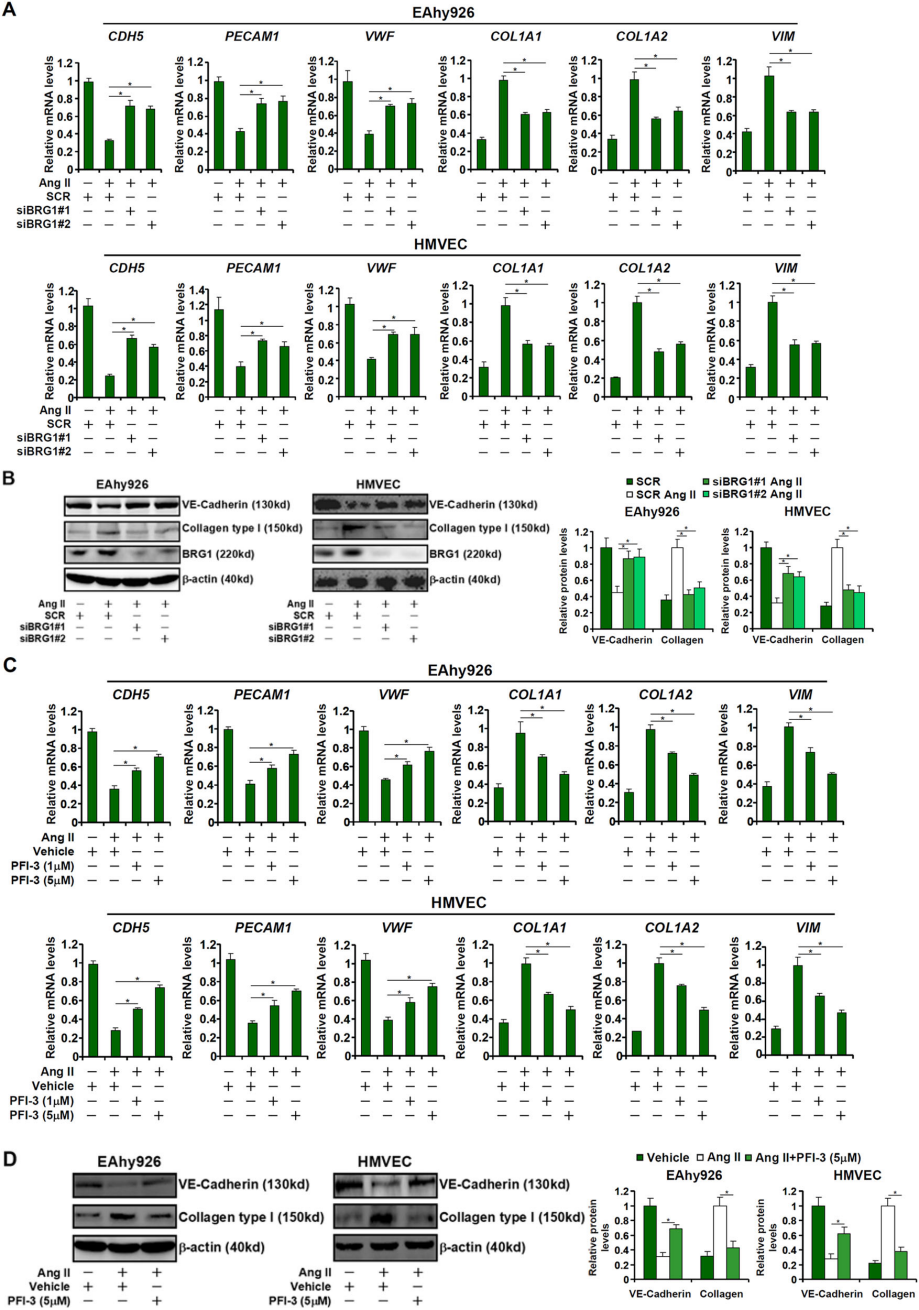
BRG1 deficiency attenuates Ang II-induced EndMT in cultured cells. **a**, **b** EAhy926 cells and HMVECs were transfected with siRNAs targeting BRG1 or scrambled siRNA (SCR) followed by treatment with Ang II (1 μM) for 48 h. Gene expression levels were examined by qPCR and Western. **c**, **d** EAhy926 cells and HMVECs were treated with Ang II (1 mM) in the presence or absence of PFI-3. Gene expression levels were examined by qPCR and Western. Data represent averages of three independent experiments and error bars represent SEM. *p < 0.05.

We first sought to determine whether BRG1 might be involved in Ang II-induced EndMT in cultured endothelial cells. To this end, endogenous BRG1 expression was silenced by small interfering RNAs (siRNAs). As shown in Fig. 1a, b, Ang II stimulation resulted in down-regulation of several signature endothelial marker genes (*CDH5*, *PECAM1*, and *VWF*) and simultaneous up-regulation of mesenchymal marker genes (*COL1A1*, *COL1A2*, and *VIM*) in immortalized vascular endothelial cells (EAhy926) as well as primary human microvascular endothelial cells (HMVECs) indicative of an EnMT-like process. BRG1 depletion, however, partially reversed the Ang II-induced EndMT by blocking *CDH5* down-regulation and *COL1A2* up-regulation. Next, the endothelial cells were treated with Ang II in the presence or absence of a small-molecule BRG1 inhibitor (PFI-3). PFI-3 treatment antagonized Ang II induced down-regulation of *CDH5* expression and up-regulation of *COL1A2* expression in a dose-dependent manner (Fig. 1c, d). These data suggest that BRG1 may contribute to Ang II-induced EndMT in cultured cells.

### BRG1 mediates induction of SNAI2/SLUG expression by Ang II

**Fig. 2:**
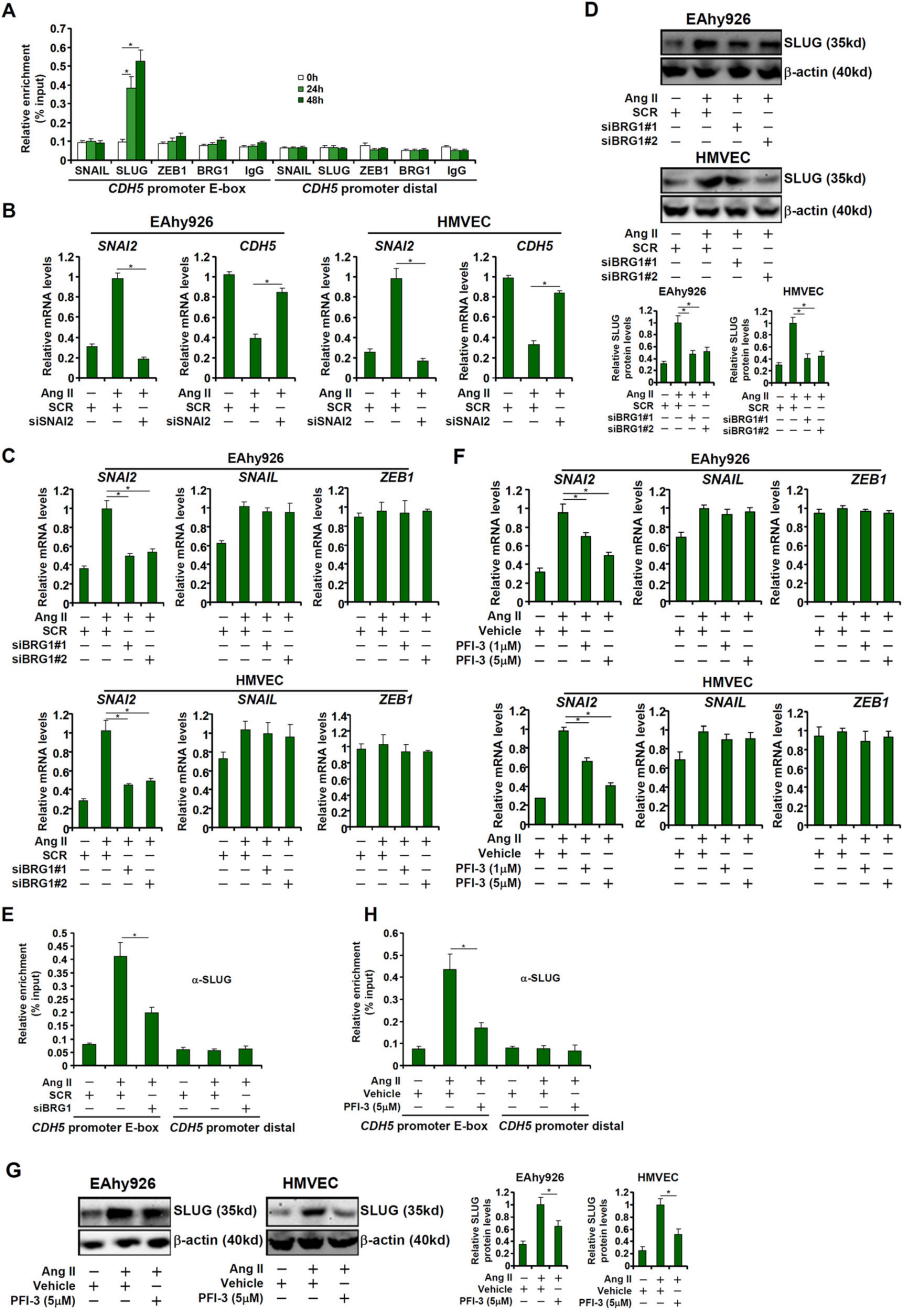
BRG1 mediates induction of SNAI2/SLUG expression by Ang II. **a** EAhy926 cells were treated with Ang II (1 μM) and harvested at indicated time points. ChIP assays were performed with indicated antibodies. **b** EAhy926 cells and HMVECs were transfected with siRNAs targeting BRG1 or scrambled siRNA (SCR) followed by treatment with Ang II (1 μM) for 48 h. Gene expression levels were examined by qPCR. **c**–**e** EAhy926 cells and HMVECs were transfected with siRNAs targeting BRG1 or scrambled siRNA (SCR) followed by treatment with Ang II (1 μM) for 48 h. SLUG expression levels were examined by qPCR and Western. ChIP assays were performed with anti-SLUG. **f**–**h** EAhy926 cells and HMVECs were treated with Ang II (1 μM) in the presence or absence of PFI-3. SLUG expression levels were examined by qPCR and Western. ChIP assays were performed with anti-SLUG. Data represent averages of three independent experiments and error bars represent SEM. *p < 0.05.

The zinc finger E-box family of transcription repressors, including SNAIL, SLUG, and ZEB, are implicated in the regulation of EMT and EndMT. ChIP assay showed that Ang II treatment robustly augmented occupancies of SLUG, but not SNAIL or ZEB1, on the *CDH5* promoter (Fig. 2a). Of note, no significant BRG1 binding was detected on the *CDH5* promoter, suggesting that BRG1 likely contributed to Ang II-induced *CDH5* trans-repression indirectly. RNAi-mediated knockdown of SLUG (encoded by *SNAI2*) normalized *CDH5* expression in endothelial cells (Fig. 2b). On the contrary, over-expression of SLUG circumvented the deficiency in BRG1 expression (Fig. S1) or activity (Fig. S2) to partially restore Ang II-induced *CDH5* repression without altering *COL1A2* expression, confirming that BRG1 may rely on SLUG to repress endothelial cell marker genes. We thus hypothesized BRG1 may directly activate *SNAI2*, which subsequently turns off *CDH5* transcription. Indeed, qPCR (Fig. 2c) and Western blotting (Fig. 2d) showed that Ang II treatment markedly up-regulated *SNAI2* expression; BRG1 knockdown by siRNAs suppressed *SNAI2* induction. Of note, BRG1 knockdown did not alter the expression of *SNAIL* and *ZEB1* (Fig. 2c). Consistent with the changes in SLUG expression, ChIP assay demonstrated that Ang II treatment increased SLUG recruitment to the *CDH5* promoter, which was blunted by BRG1 depletion (Fig. 2e). Likewise, BRG1 inhibition by PFI-3 blocked Ang II-induced SLUG expression as well as its recruitment to the *CDH5* promoter (Fig. 2f-h). Again, neither *SNAIL* expression nor *ZEB1* expression was altered by BRG1 inhibition (Fig. 2f).

### BRG1 activates SNAI2 transcription by interacting with Sp1

**Fig. 3:**
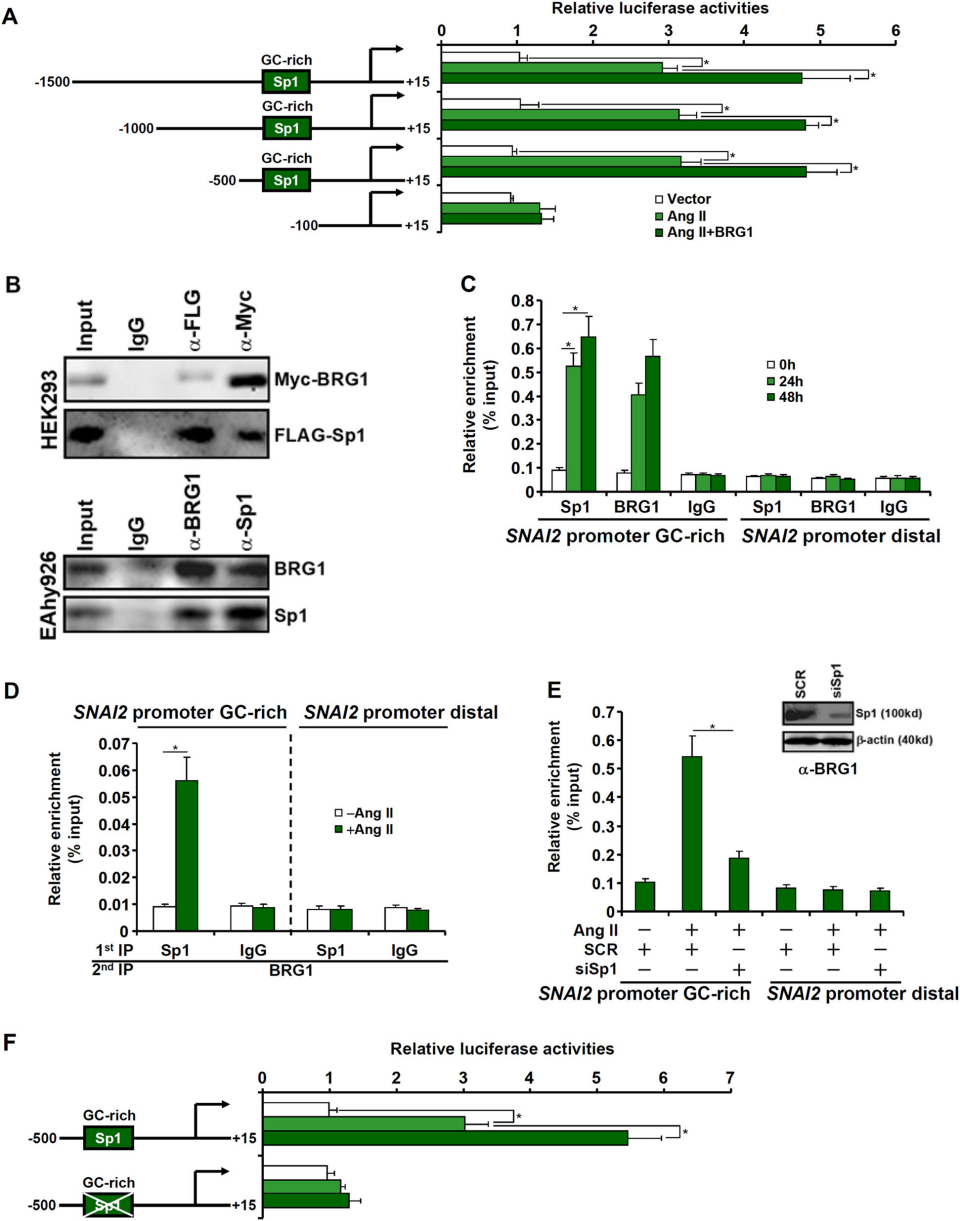
BRG1 activates SNAI2 transcription by interacting with Sp1. **a** SNAI2 promoter-luciferase constructs were transfected into EAhy926 cells with or without BRG1 followed by treatment with Ang II (1 μM). Luciferase activities were normalized by both protein concentration and GFP fluorescence. **b** (Upper panel) Nuclear lysates from HEK293 cells were immunoprecipitated with indicated antibodies. (Bottom panel) Nuclear lysates from EAhy926 cells were immunoprecipitated with indicated antibodies. **c** EAhy926 cells were treated with Ang II (1 μM) and harvested at indicated time points. ChIP assays were performed with indicated antibodies. **d** EAhy926 cells were treated with or without Ang II (1 μM) for 48 h. Re-ChIP assays were performed with indicated antibodies. **e** EAhy926 cells were transfected with siRNAs targeting Sp1 or scrambled siRNA (SCR) followed by treatment with Ang II (1 μM) for 48 h. ChIP assays were performed with indicated antibodies. **f** Wild type and mutant SNAI2 promoter-luciferase constructs were transfected into EAhy926 cells with or without BRG1 followed by treatment with Ang II (1 μM). Luciferase activities were normalized by both protein concentration and GFP fluorescence. Data represent averages of three independent experiments and error bars represent SEM. *p < 0.05.

We next explored the mechanism by which BRG1 regulates *SNAI2* transcription. A series of *SNAI2* promoter-luciferase constructs were transfected into endothelial cells with or without BRG1 followed by Ang II treatment. As shown in Fig. 3a, Ang II treatment activated the *SNAI2* promoter activity and BRG1 over-expression further enhanced the activation. The responsiveness to Ang II treatment and BRG1 over-expression was lost on the *SNAI2* promoter when the progressively inward deletion extended to −100 relative to the transcription start site suggesting that an Ang II/BRG1 response element might be located between −500 and −100 (Fig. 3a). Because a conserved Sp1-binding site (GC-rich) could be found within this region of the *SNAI2* promoter, we proposed that BRG1 might interact with Sp1 to activate *SNAI2* transcription. We performed the following experiments to test this proposal. First, FLAG-tagged Sp1 and Myc-tagged BRG1 were co-transfected into HEK293 cells. Immunoprecipitation assay showed that Sp1 and BRG1 formed a complex in the cells (Fig. 3b, upper panel). In addition, endogenous BRG1 and Sp1 were also discovered to be in the same complex (Fig. 3b, bottom panel). ChIP assay confirmed that in response to Ang II treatment both Sp1 and BRG1 started to occupy the same GC-rich region of the *SNAI2* promoter (Fig. 3c). More importantly, Ang II stimulation promoted the assembly of an Sp1–BRG1 complex on the *SNAI2* promoter as evidenced by Re-ChIP assay (Fig. 3d). The reliance of BRG1 on Sp1 to activate *SNAI2* transcription was further supported by the observation that depletion of Sp1 by siRNA drastically reduced BRG1 binding on the *SNAI2* promoter (Fig. 3e). Finally, mutation of the Sp1 motif (GC-rich region) on the *SNAI2* promoter completely abrogated its activation by Ang II plus BRG1 (Fig. 3f).

### BRG1 interacts with SRF to activate COL1A2 transcription

**Fig. 4:**
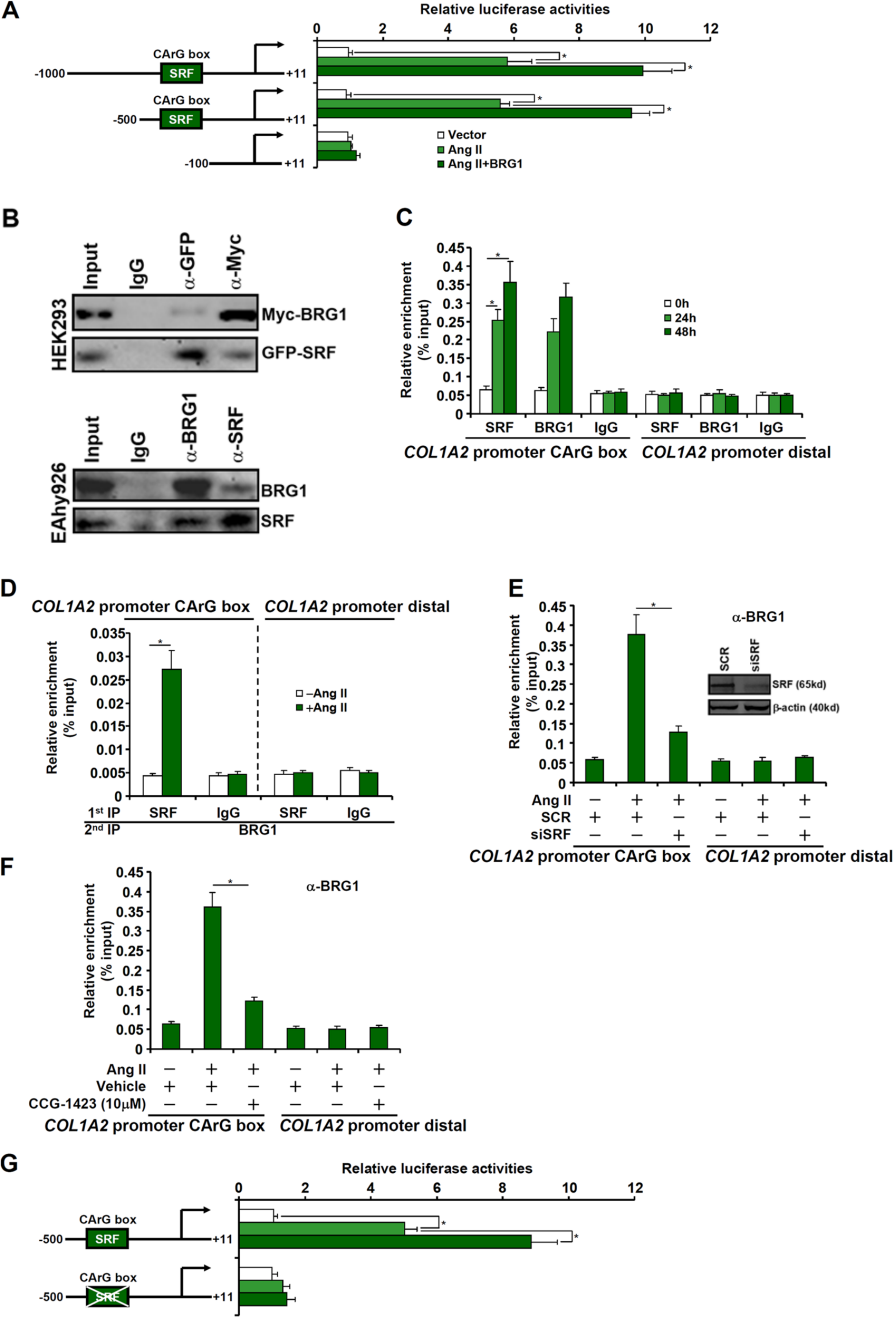
BRG1 interacts with SRF to activate COL1A2 transcription. **a** COL1A2 promoter-luciferase constructs were transfected into EAhy926 cells with or without BRG1 followed by treatment with Ang II (1 μM). Luciferase activities were normalized by both protein concentration and GFP fluorescence. **b** (Upper panel) Nuclear lysates from EAhy926 cells were immunoprecipitated with indicated antibodies. (Bottom panel) Nuclear lysates from EAhy926 cells were immunoprecipitated with indicated antibodies. **c** EAhy926 cells were treated with Ang II (1 μM) and harvested at indicated time points. ChIP assays were performed with indicated antibodies. **d** EAhy926 cells were treated with or without Ang II (1 μM) for 48 h. Re-ChIP assays were performed with indicated antibodies. **e** EAhy926 cells were transfected with siRNAs targeting SRF or scrambled siRNA (SCR) followed by treatment with Ang II (1 μM) for 48 h. ChIP assays were performed with indicated antibodies. **f** EAhy926 cells and HMVECs were treated with Ang II (1 μM) in the presence or absence of CCG-1423. ChIP assays were performed with indicated antibodies. **g** Wild type and mutant COL1A2 promoter-luciferase constructs were transfected into EAhy926 cells with or without BRG1 followed by treatment with Ang II (1 μM). Luciferase activities were normalized by both protein concentration and GFP fluorescence. Data represent averages of three independent experiments and error bars represent SEM. *p < 0.05.

We next examined the possibility that BRG1 might directly activate *COL1A2* transcription. To this end, human *COL1A2* promoter-luciferase constructs of different lengths were transfected into endothelial cells. Ang II treatment stimulated the *COL1A2* promoter activities, which were further augmented by BRG1 over-expression (Fig. 4a). Activation of the *COL1A2* promoter by Ang II and BRG1 was indiscernible once the deletion went beyond −500 relative to the transcription start site. Small et al. have previously identified a conserved binding site (CArG box) for the sequence-specific transcription factor SRF between −500 and −100 of the *COL1A2* promoter. We therefore investigated a potential interplay between SRF and BRG1 in mediating Ang II-induced *COL1A2* transcription in endothelial cells. Co-immunoprecipitation assay confirmed that BRG1 and SRF interacted with each other in HEK293 cells (Fig. 4b, upper panel) and in EAhy926 cells (Fig. 4b, bottom panel). When the endothelial cells were exposed to Ang II, SRF and BRG1 were recruited to the proximal *COL1A2* promoter with similar kinetics (Fig. 4c). In addition, Ang II treatment promoted the formation of an SRF–BRG1 complex on the *COL1A2* promoter (Fig. 4d). SRF knockdown by small-interfering RNA (Fig. 4e) or inhibition by a small-molecule chemical (CCG-1423, Fig. 4f) compromised BRG1 recruitment to the *COL1A2* promoter. Functionally, disruption of the CArG box by mutagenesis rendered the *COL1A2* promoter irresponsive to Ang II treatment and BRG1 over-expression (Fig. 4g).

### BRG1 facilitates the bindings of Sp1 and SRF by evicting histones from chromatin

**Fig. 5:**
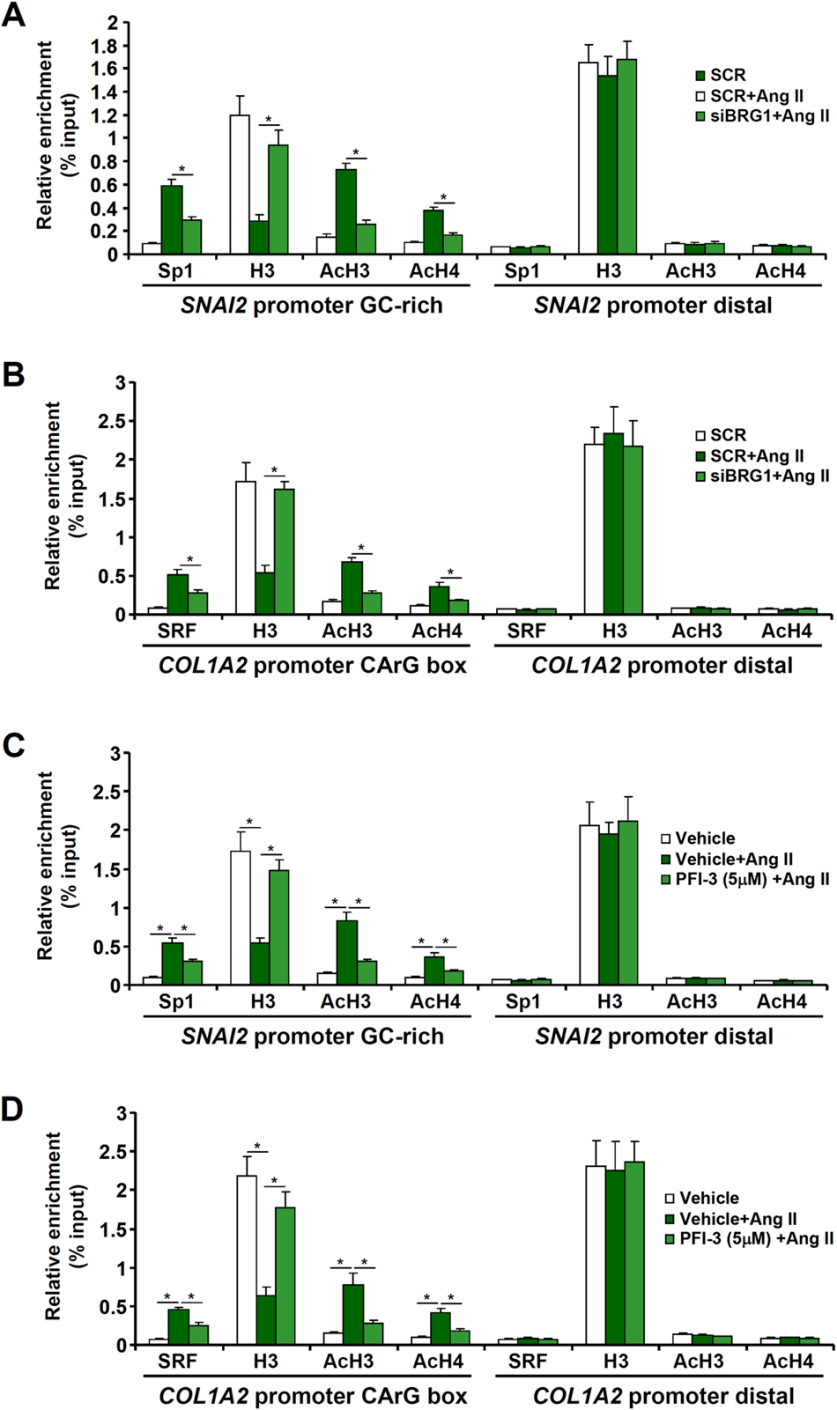
BRG1 facilitates the bindings of Sp1 and SRF by evicting histones from chromatin. **a**, **b** EAhy926 cells were transfected with siRNAs targeting BRG1 or scrambled siRNA (SCR) followed by treatment with Ang II (1 μM) for 48 h. ChIP assays were performed with indicated antibodies. **c**, **d** EAhy926 cells were treated with Ang II (1 μM) in the presence or absence of PFI-3 for 48 h. ChIP assays were performed with indicated antibodies. Data represent averages of three independent experiments and error bars represent SEM. *p < 0.05.

Sequence-specific transcription factors rely on effective chromatin remodeling to access their binding motifs. We next evaluated whether BRG1 deficiency might influence the activities of Sp1 and SRF during Ang II-induced EndMT. ChIP assays revealed that BRG1 depletion (Fig. 5a, b) or inhibition (Fig. 5c, d) was concordant with weakening of Sp1 binding on the *SNAI2* promoter and dampening of SRF binding on the *COL1A2* promoter. Ang II treatment resulted in reduced levels of histones associated with the *SNAI2* promoter (Fig. 5a, c) and the *COL1A2* promoter (Fig. 5b, d), consistent with a loosened chromatin structure. BRG1 deficiency, however, restored the abundance of histones on the *SNAI2* promoter and the *COL1A2* promoter. In addition, Ang II treatment led to accumulation of acetylated histone H3 and H4 on the *SNAI2* promoter (Fig. 5a, c) and the *COL1A2* (Fig. 5b, d) promoter, which was dampened by BRG1 deficiency. Thus, it appeared that BRG1 might have contributed to Ang II-induced EndMT by modulating the chromatin structure to aid the binding of transcription factors.

### Endothelial-specific BRG1 deletion attenuates Ang II-induced EndMT and cardiac fibrosis in mice

**Fig. 6:**
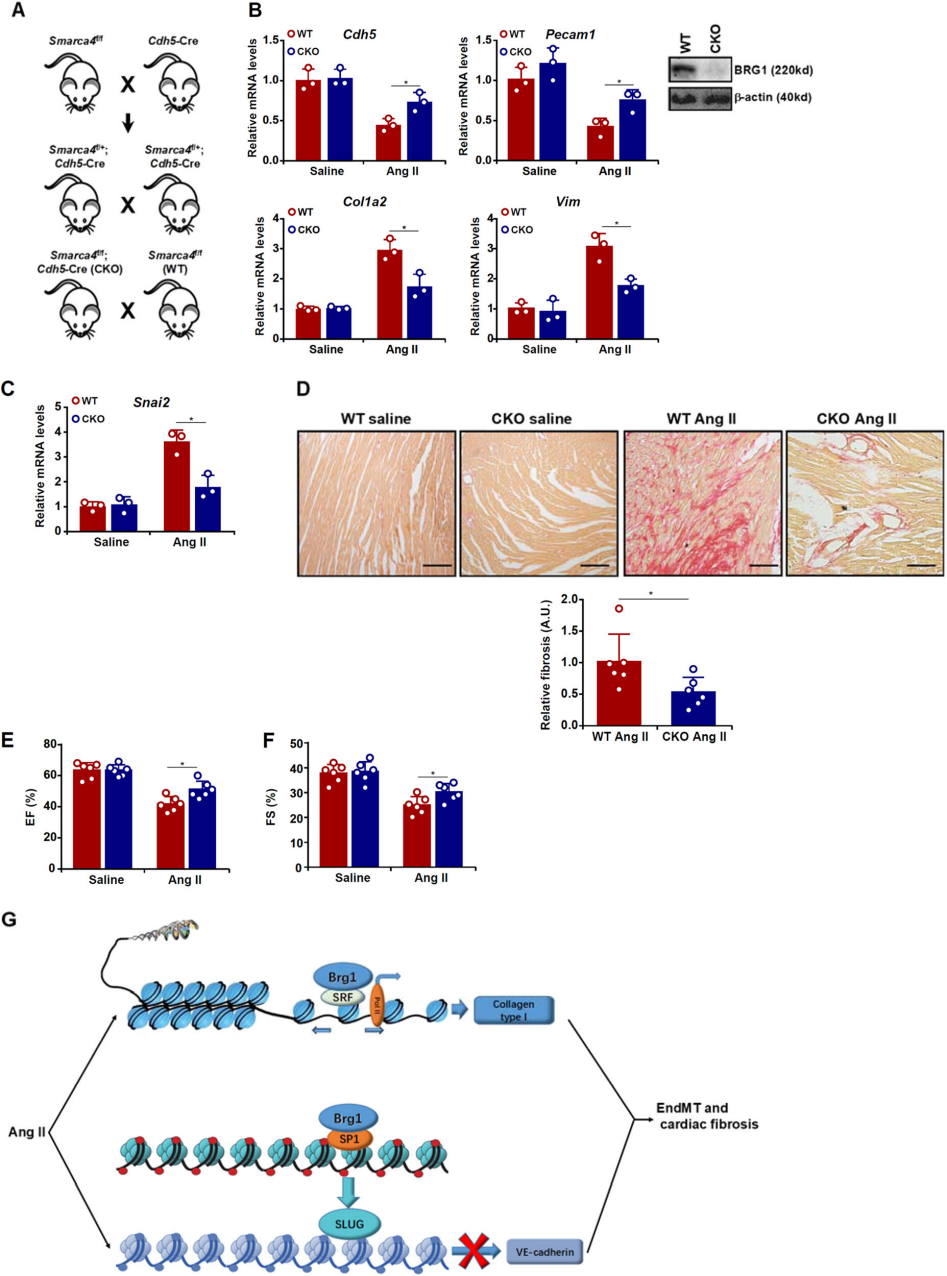
Endothelial-specific BRG1 deletion attenuates Ang II-induced EndMT and cardiac fibrosis in mice. Endothelial-specific BRG1 knockout (CKO) mice and wild type (WT) mice were induced to develop cardiac fibrosis by chronic Ang II infusion as described in the “Methods” section. **a** A scheme of crossbreeding that generates the CKO mice. **b** Primary cardiac microvascular endothelial cells were isolated and gene expression levels were examined by qPCR. Inset, BRG1 knockout efficiency was verified by Western. **c** Primary cardiac microvascular endothelial cells were isolated and SNAI2 levels were examined by qPCR. *N* = 3 mice for each group. **d** Picrosirius red staining and quantification. *N* = 6 mice for each group. **e**, **f** Echocardiographic measurements of EF and FS values. *N* = 6 mice for each group. **g** A schematic model.

We finally attempted to tackle the question as to whether the data obtained from cell culture could be extrapolated to an animal model. BRG1 was specifically deleted in vascular endothelial cells in mice by *Cdh5*-Cre driven removal of the floxed *Smarca4* allele (Fig. 6a). Both endothelial BRG1 CKO mice and the control (WT) mice were subjected to chronic Ang II infusion for 4 weeks to induce cardiac fibrosis. Quantitative PCR analysis performed in primary cardiac microvascular endothelial cells isolated from the mice revealed that Ang II infusion led to a decrease in *Cdh5* expression and a simultaneous increase in *Col1a2* expression suggesting that the EndMT-like process observed in cell culture could be replicated in the murine hearts (Fig. 6b). More important, *Snai2* expression was lower in the primary microvascualr endothelial cells isolated from the Ang II-infused CKO hearts than from the Ang II-infused WT hearts (Fig. 6c). Changes in gene expression patterns were consistent with the observation that cardiac fibrosis was attenuated in the CKO mice as evidenced by weaker picrosirius red staining of collagenous tissues in the heart (Fig. 6d). In accordance, suppression of heart function, indicated by echocardiographic measurements of ejection fraction (EF, Fig. 6e) and fractional shortening (FS, Fig. 6f) following chronic Ang II infusion was alleviated in the CKO mice compared to the WT mice.

## Discussion

Endothelial plasticity is essential to embryonic development. Aberrant and inadvertent activation of EndMT, however, is associated with the pathogenesis of a myriad of human diseases including cardiac fibrosis^45–47^. Here we describe a novel epigenetic pathway in which Ang II-induced EndMT can be ascribed to dual roles of BRG1, a chromatin remodeling protein (Fig. 6g). BRG1 on the one hand cooperates with Sp1 to activate the transcription of SLUG (*SNAI2*), which functions as a repressor of endothelial gene (*CDH5*) transcription. On the other hand, BRG1 interacts with SRF to activate the transcription of collagen type I (*COL1A2*), a mesenchymal marker. More important, endothelial-specific deletion of BRG1 significantly ameliorated Ang II-induced EndMT and cardiac fibrosis in mice. It should be noted that there are a few caveats with regard to the proposed model. First, it remains unknown why BRG1 selectively activates the transcription of SLUG (*SNAI2*), but not SNAIL or ZEB1, to promote the loss of endothelial markers because SNAIL^48^ and ZEB1^49^ have been shown to mediate EndMT in different settings. This preference could be attributed to a combination of specific cell types, stimuli, and sequence-specific transcription factors that recruit BRG1. For instance, ZEB1, but not SNAIL or SLUG, is activated by Wnt signaling to promote EMT in the kidneys^50^. SNAIL, but not ZEB1 or SLUG, is found to be specifically up-regulated in poorly differentiated and mesenchymal-like breast cancer cells^51^ and colon cancer cells^52^. The redundancies among different EndMT/EMT factors are an intriguing issue^53^ and our data suggest that BRG1 might be a contributing factor but this hypothesis deserves further attention. Second, BRG1 may contribute to Ang II-induced EndMT via alternative mechanisms. Accumulation of reactive oxygen species (ROS) is considered a key driving force of EndMT^54^. It has been found that BRG1 modulates intracellular ROS levels by transcriptionally activating the genes involved in ROS synthesis^16,27^. In fact, we have previously shown that activation of NADPH oxidase 4 (NOX4) gene transcription in endothelial cells by BRG1 may be responsible for TGF-β-induced EndMT and liver fibrosis^40^. The latter observation is not at variance but rather consistent with the present data because Ang II stimulation can trigger ROS accumulation and simultaneous up-regulation of collagen type I expression^55,56^. Third, BRG1-mediated EndMT may not fully account for Ang II-induced cardiac fibrosis in vivo. Chronic inflammation is one of the major culprits for cardiac fibrosis^57,58^. We have recently shown that BRG1 deficiency in endothelial cells attenuates inflammation in the vessel wall^14,59^ and in the kidneys^60^, which can be partly explained by the fact that BRG1 activates the transcription of adhesion molecules (e.g., *ICAM-1*) and chemokines (e.g., *CCL2*) to promote macrophage trafficking. Alternatively, BRG1 may regulate the expression of endothelial-derived humoral factors to influence cardiac fibrosis. For instance, BRG1 has been shown to activate the transcription of endothelin (*ET-1*), a pro-fibrogenic factor^61^, and repress the transcription of eNOS (*NOS3*), an anti-fibrogenic factor^62^, in endothelial cells. Therefore, attenuation of cardiac fibrosis in the CKO mice may be construed as a consequence of skewed balance between endothelial-derived pro-fibrogenic and anti-fibrogenic factors. It should be pointed out that BRG1 may be able to directly repress *CDH5* transcription to promote EMT/EndMT-like processes. Sánchez-Tilló E et al. have reported that BRG1 can form a complex with ZEB1 to directly bind to the *CDH5* promoter to repress its transcription and promote β-catenin nuclear trans-location in several different cancer cells^63^. The discrepancies in our observations and those by Sánchez-Tilló E et al. likely reflect the complex nature regarding BRG1-mediated transcription regulation in different cells and different circumstances. A ChIP-seq analysis with anti-BRG1 antibodies aiming to examine the dynamic association of BRG1 with target promoters during EndMT will likely provide additional mechanistic insight on the precise role BRG1 plays in regulating this process. Finally, Ang II not only promotes cardiac remodeling and fibrosis but vascular remodeling and hypertension. Ang II-induced endothelial dysfunction including altered endothelial gene expression is noted in model animals and in humans. Therefore, it would be of great interest to determine whether BRG1 deficiency normalizes blood pressure in the model as described in Fig. 6.

We show here that BRG1 contributes to Ang II-induced trans-activation of *SNAI2* and *COL1A2* by modulating histone status. Eviction of histones from the chromatin, as observed here, during nucleosome mobilization is one of the better understood functionalities of BRG1. For instance, regulation of *eNOS* transcription in endothelial cells exposed to hypoxia involves BRG1-mediated histone eviction from the *eNOS* promoter^64^. We have previously shown that eviction of histones from the *CRP* promoter by BRG1 underlies its trans-activation by free fatty acids in hepatocytes^43^. Of note, recent studies have portrayed a scenario wherein BRG1 relies on its interactions with histone-modifying enzymes to regulate the transcription of target genes. The observation that the status of histone H3/H4 acetylation is associated with *SNAI2*/*COL1A2* transcription compels a natural question as to whether specific histone acetyltransferases may participate in EndMT and cardiac fibrosis through BRG1. A series of independent studies have shown that E1A-associated protein 300 (p300), a well-documented binding partner for BRG1, regulates EndMT by potentiating the TGF-β-signaling pathway^65^. Although not investigated in the present study, other histone-modifying enzymes that play a role in EndMT, including histone deacetylase 3 (HDAC3)^66,67^, histone H3K4 methyltransferase WDR5^68^, histone H3K27 methyltransferase EZH2^69^, and histone demethylase JMJD2B^40^, can all interact with BRG1 in various settings. A comprehensive mapping of BRG1-dependent histone modifications on a genomewide scale will likely provide novel insights on the epigenetic regulation of EndMT.

BRG1 relies on sequence-specific transcription factors to participate in locus-specific transcriptional regulation and, by extension, pathogenesis of human diseases. Our data indicate that BRG1 is recruited by Sp1 and SRF to the *SNAI2* promoter and *COL1A2* promoter, respectively, raising the possibility that targeting Sp1 and/or SRF in endothelial cells may phenocopy the BRG1 CKO mice in the model of Ang II-induced cardiac fibrosis. Both Sp1 and SRF have been demonstrated to contribute to myofibroblast maturation in the context of cardiac fibrosis although cell-specific roles remain undetermined^70,71^. We have previously shown that BRG1 mediates EndMT induced by TGF-β, ROS, and hypoxia^22,72^. In light of our new findings as summarized here, targeting the endothelial BRG1–Sp1–SRF cluster may be considered as a reasonable approach in the intervention of cardiac fibrosis.

## Supplementary information

Supplementary Figure legendsn

Supplementary Figure S1

Supplementary Figure S2
