# Supplementary material for: Dual roles of chromatin remodeling protein BRG1 in angiotensin II-induced endothelial–mesenchymal transition
Source: Cell Death Dis. 2020 Jul 18;11(7):549. doi: 10.1038/s41419-020-02744-y (PMC7368857; doi:10.1038/s41419-020-02744-y)
Supplement: Supplementary file 1 — Supplementary Figure legendsn [file 41419_2020_2744_MOESM1_ESM.docx]

Supplementary Figure legends

**Fig.S1**: (**A, B**) EAhy926 cells were transfected with siRNA targeting BRG1 in the presence or absence of a SLUG expression construct followed by treatment with Ang II. Gene expression levels were examined by qPCR and Western.

**Fig.S2:** (**A, B**) EAhy926 cells were treated with Ang II and PFI-3 in the presence or absence of a SLUG expression construct. Gene expression levels were examined by qPCR and Western.
