# Supplementary figures and images for: Dual roles of chromatin remodeling protein BRG1 in angiotensin II-induced endothelial–mesenchymal transition
Source: Cell Death Dis. 2020 Jul 18;11(7):549. doi: 10.1038/s41419-020-02744-y (PMC7368857; doi:10.1038/s41419-020-02744-y)

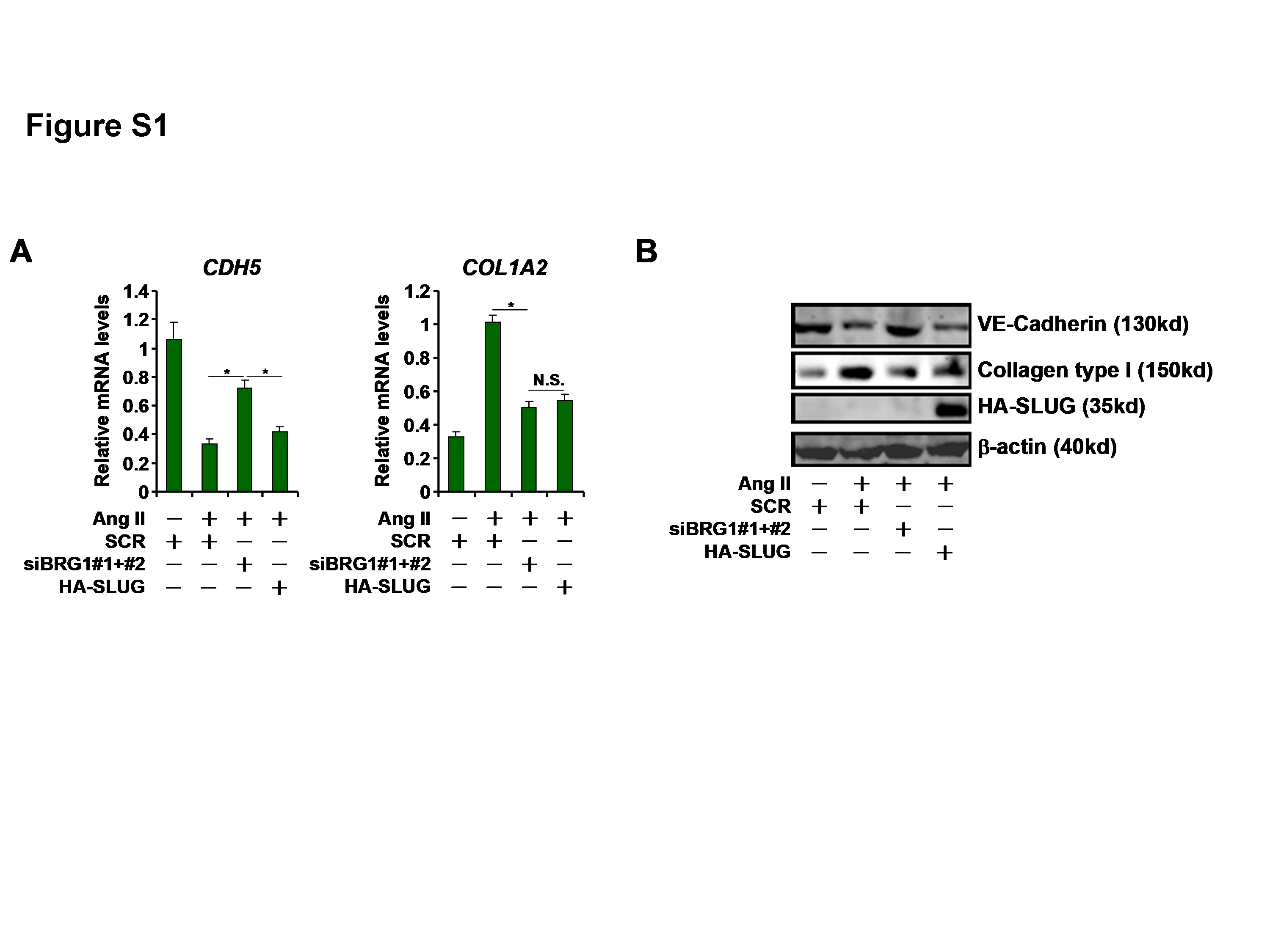

Supplement: Supplementary file 2 — Supplementary Figure S1 [file 41419_2020_2744_MOESM2_ESM.tif]

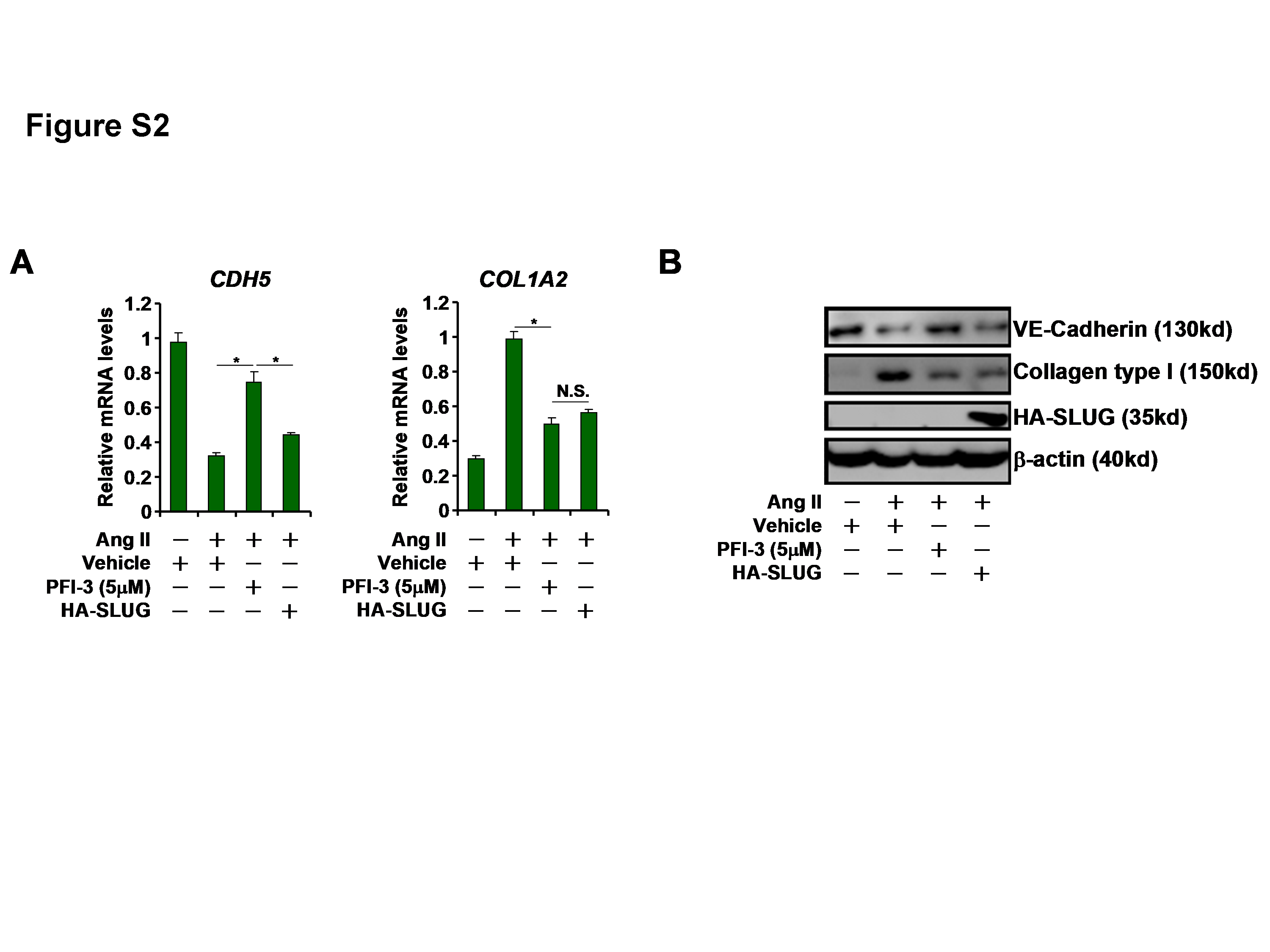

Supplement: Supplementary file 3 — Supplementary Figure S2 [file 41419_2020_2744_MOESM3_ESM.tif]
